# Adherence to dietary guidelines in relation to visceral fat and liver fat in middle-aged men and women: the NEO study

**DOI:** 10.1038/s41366-019-0441-x

**Published:** 2019-08-28

**Authors:** Esther van Eekelen, Anouk Geelen, Marjan Alssema, Hildo J. Lamb, Albert de Roos, Frits R. Rosendaal, Renée de Mutsert

**Affiliations:** 1 Department of Clinical Epidemiology, Leiden University Medical Center, Leiden, The Netherlands; 2 Department of Human Nutrition, Wageningen University & Research, Wageningen, The Netherlands; 3 Unilever Research and Development, Vlaardingen, The Netherlands; 4 Department of Epidemiology and Biostatistics, Amsterdam Public Health research institute, VU University Medical Center, Amsterdam, The Netherlands; 5 Department of Radiology, Leiden University Medical Center, Leiden, The Netherlands

**Keywords:** Risk factors, Epidemiology

## Abstract

**Background:**

It is unclear to what extent adherence to dietary guidelines may specifically affect visceral fat and liver fat. We aimed to study the association between the Dutch Healthy Diet Index (DHD-index) and total body fat, visceral adipose tissue (VAT) and hepatic triglyceride content (HTGC) in middle-aged men and women.

**Design:**

In this cross-sectional study, VAT was assessed by magnetic resonance imaging (MRI) in 2580 participants, and HTGC by proton-MR spectroscopy in 2083 participants. Habitual dietary intake and physical activity were estimated by questionnaire. Adherence to the current Dutch dietary guidelines was estimated by the 2015 DHD-index score based on the thirteen components (vegetables, fruit, wholegrain products, legumes, nuts, dairy, fish, tea, liquid fats, red meat, processed meat, sweetened beverages, and alcohol). The DHD-index ranges between 0 and 130 with a higher score indicating a healthier diet. We used linear regression to examine associations of the DHD-index with VAT and HTGC, adjusted for age, smoking, education, ethnicity, basal metabolic rate, energy restricted diet, menopausal state, physical activity, total energy intake, and total body fat. We additionally excluded the components one by one to examine individual contributions to the associations.

**Results:**

Included participants (43% men) had a mean (SD) age of 56 (6) years and DHD-index score of 71 (15). A 10-point higher DHD-index score was associated with 2.3 cm^2^ less visceral fat (95% CI; −3.5; −1.0 cm^2^) and less liver fat (0.94 times, 95% CI; 0.90; 0.98). Of all components, exclusion of dairy attenuated the associations with TBF and VAT.

**Conclusions:**

Adherence to the dietary guidelines as estimated by the DHD-index was associated with less total body fat, and with less visceral and liver fat after adjustment for total body fat. These findings might contribute to better understanding of the mechanisms underlying associations between dietary habits and cardiometabolic diseases.

## Introduction

The prevalence of obesity is increasing worldwide. In particular abdominal obesity is a well-established risk factor for the metabolic syndrome, diabetes mellitus, and cardiovascular diseases [1, 2]. The excess risk of abdominal obesity is hypothesized to be due to the accumulation of fat in the visceral area and in and around the organs (ectopic fat) [2], such as in the liver. Visceral adipose tissue (VAT) and hepatic triglyceride content (HTGC) have been associated with insulin resistance, metabolic risk factors, and cardiovascular disease [3–6]. Due to these multiple health-related consequences, visceral fat and liver fat are important targets for battling cardiometabolic diseases.

Together with physical activity, diet is an essential modifiable risk factor for obesity and obesity-related chronic diseases [7]. Recently, dietary guidelines have started to shift from nutrient-based to food-based and dietary patterns, as humans do not consume separate nutrients but rather combinations of foods [8–10]. Also, some nutrient effects might be too small to detect separately [11] and different nutrients might be strongly correlated or even interact with each other, making it hard to disentangle their effects. As nutrient intakes are often associated with certain dietary patterns, analyses including only one nutrient might therefore be confounded by dietary patterns [9].

Numerous dietary indices of adherence to a healthy diet have been developed over the last two decades, among which the (Alternative) Healthy Eating Index (HEI) [12], the Healthy Diet Indicator (HDI) [13], and the Diet Quality Index (DQI) [14]. The HDI has been associated with both all-cause and cardiovascular disease mortality and the DQI with circulatory disease mortality in women [15]. The HEI has also been associated with obesity [16], which might be an underlying mechanism for the association between the HEI and cardiovascular disease, as shown in previous research [15].

However, it remains unknown whether adherence to dietary guidelines has specific effects on visceral fat and HTGC or merely on overall body fat. Therefore, we aimed to study the association between the Dutch Healthy Diet Index (DHD-index) and total body fat (TBF), VAT and liver fat. In addition, we explored which components of the DHD-index contributed the most to the associations with TBF, visceral fat, and liver fat.

## Methods

### Study design and study population

The Netherlands Epidemiology of Obesity (NEO) study is a population-based prospective cohort study in 6671 individuals aged 45–65 years, with an oversampling of persons with a BMI of 27 kg/m^2^ or higher. Detailed information about the study design and data collection has been described elsewhere [17]. Men and women aged between 45 and 65 years with a self-reported BMI of 27 kg/m^2^ or higher living in the greater area of Leiden (in the West of The Netherlands) were eligible to participate in the NEO study. In addition, all inhabitants aged between 45 and 65 years from one municipality (Leiderdorp) were invited irrespective of their BMI, allowing for a reference distribution of BMI.

Participants visited the NEO study center of the Leiden University Medical Center after an overnight fast. Prior to the NEO study visit, participants completed a questionnaire about demographic, lifestyle, and clinical information, in addition to a food frequency questionnaire (FFQ). At the study center, the participants completed a screening form, asking about anything that might create a health risk or interfere with magnetic resonance imaging (MRI) (most notably metallic devices, claustrophobia, or a body circumference of more than 1.70 m). Of the participants who were eligible for MRI, ~35% were randomly selected to undergo direct assessment of abdominal fat.

The present study is a cross-sectional analysis of the baseline measurements. We excluded participants with implausibly low or high total energy intake (<600 kcal or >5000 kcal/day), which are somewhat less conservative cutoff points for high energy intake than other cohort studies [12] because of our smaller sample size. Moreover, we excluded participants with missing data on dietary intake or potential confounding factors. For the analyses on liver fat, we additionally excluded participants who consumed more than four standard units of alcohol per day.

The study was approved by the medical ethics committee of the Leiden University Medical Center and conducted according to the declaration of Helsinki. All participants gave written informed consent.

### Data collection

On the questionnaire, participants reported ethnicity by self-identification in eight categories which we grouped into white (reference) and other. Tobacco smoking was reported in the three categories current, former, and never smoking (reference). The highest level of education was reported in ten categories according to the Dutch education system and grouped into high (including higher vocational school, university, and postgraduate education) versus low education (reference). Participants reported their medical history of diabetes and cardiovascular diseases. Preexisting cardiovascular disease was defined as myocardial infarction, angina, congestive heart failure, stroke, or peripheral vascular disease. Body weight was measured without shoes and 1 kg was subtracted from the body weight. Percent body fat was estimated using bioelectrical impedance analysis (BIA) with the Tanita foot-to-foot (FF) BIA system TBF-300A Body Composition Analyzer [18]. BMI was calculated by dividing the weight in kilograms by the height in meters squared. Menopausal state was categorized in pre- and post-menopausal state according to information on ovariectomy, hysterectomy, and self-reported state of menopause in the questionnaire. Basal metabolic rate was calculated based on age, sex, height, and weight according to the Mifflin–St Jeor equation [19]. Participants reported the frequency, duration, and intensity of their habitual physical activity during leisure time using the Short Questionnaire to Assess Health-enhancing physical activity, which was expressed in hours per week of metabolic equivalents (MET h/week) [20].

### Dutch Healthy Diet Index

Habitual dietary intake of all participants was estimated using a semiquantitative self-administered 125-item FFQ [21, 22]. In this questionnaire, participants reported their frequency of intake of foods during the past month (times per day, week, month, never). This was combined with the assessment of serving size (spoons of potatoes, pieces of fruit, etc). Dietary intake of nutrients and total energy was estimated using the Dutch Food Composition Table (NEVO-2011).

Based on the FFQ, we calculated the DHD-index for each participant, which is a continuous score and represents the adherence to the Dutch Guidelines for Healthy Diet of 2015 as described by the Health Council of the Netherlands and originally consists of fifteen components [23]. Every index component has a maximum of 10 points, depending on the cutoff as described by the guidelines for each component: vegetables (≥200 g per day), fruit (≥200 g per day), wholegrain products (ratio of whole grains to refined grains ≥11), legumes (≥10 g per day), unsalted nuts (≥15 g per day), dairy (between 300 and 450 g per day), fish (≥15 g per day), tea (≥450 g per day), replacing hard fats by liquid fats (ratio of liquid to solid fats ≥13), coffee (consumption of only filtered coffee), red meat (≤45 g per day), processed meat (0 g per day), sweetened beverages (0 g per day), alcohol (≤10 g per day), and salt (≤1.9 g of sodium per day). Assessing the adherence to these guidelines is based on the five types of components in the DHD-index: (1) adequacy components (minimum consumption recommended, e.g., vegetables, fruit, wholegrain products, legumes, and nuts), (2) moderation components (limited consumption recommended, e.g., red meat, processed meat, sweetened beverages, alcohol, and salt), (3) optimum components (consumption between certain limits recommended, e.g., dairy), (4) qualitative components (recommended consumption depending on quality of product, e.g., coffee), and (5) ratio components (a certain ratio of consumption is recommended, e.g., fats and oils and wholegrain products) [23]. As a result, the total score can range between 0 and 150. A higher score means a better adherence to the 2015 Dutch Guidelines for a Healthy Diet. For the present study, we used an adapted version of the DHD-index with thirteen components instead of the original fifteen because we were not able to estimate the two components consumption of unfiltered coffee, and of sodium on the basis of the FFQ used in our study. As a result, the DHD-index in our study ranges between 0 and 130.

### Visceral fat area and hepatic triglyceride content by imaging techniques

Imaging was performed on a 1.5 Tesla MR system (Philips Medical Systems, Best, the Netherlands). VAT was quantified by a turbo spin echo imaging protocol using MRI. At the level of the fifth lumbar vertebra, three transverse images each with a slice thickness of 10 mm were obtained during a breath-hold. Visceral fat area was converted from the number of pixels to centimeters squared using in-house-developed software (MASS, Medis, Leiden, the Netherlands) and the average of three slices was used in the analyses [17].

HTGC was quantified by proton-MR spectroscopy (^1^H-MRS) of the liver [24]. An 8 ml voxel positioned in the right lobe of the liver, avoiding gross vascular structures and adipose tissue depots. Sixty-four averages were collected with water suppression. Spectra were obtained with an echo time of 26 ms and a repetition time of 3000 ms. Data points (1024) were collected using a 1000 Hz spectral line. Without changing any parameters, spectra without water suppression, with a repetition time of 10 s, and with four averages were obtained as an internal reference . ^1^H-MRS data were fitted using Java-based magnetic resonance user interface software (jMRUI version 2.2, Leuven, Belgium), as described previously [25]. HTGC relative to water was calculated the sum of signal amplitudes of methyl and methylene divided by the signal amplitude of water and then multiplied by 100.

### Statistical analyses

In the NEO study there is an oversampling of persons with a BMI of 27 kg/m^2^ or higher. To correctly represent associations in the general population [26], adjustments for the oversampling of individuals with a BMI ≥ 27 kg/m^2^ were made. This was done by weighting individuals towards the BMI distribution of participants from the Leiderdorp municipality [27], whose BMI distribution was similar to the BMI distribution of the general Dutch population [28]. All results were based on weighted analyses. Consequently, the results apply to a population-based study without oversampling of individuals with a BMI ≥ 27 kg/m^2^. As a result of the weighted analyses, percentages and proportions are given instead of numbers of participants. Other baseline characteristics are expressed as mean with standard deviation.

We performed linear regression analyses with multiple models. First, we studied the association between the DHD-index (per 10 points) with overall adiposity, as measured by TBF (%). We performed both a crude model and a multivariable model adjusted for age, sex, smoking, education, ethnicity, basal metabolic rate, menopausal state, physical activity, adherence to an energy restricted diet, and total energy intake. After this, we examined the associations between the DHD-index with visceral fat and liver fat content. To examine whether the associations were specific for visceral fat and liver fat instead of merely representing effects on total adiposity, we additionally corrected for TBF in a separate model. For this, we calculated variance inflation factors (VIFs) to check for multicollinearity in our models between visceral fat or liver fat and TBF. The VIF values were below 10 in all models and were considered appropriate. Correlation coefficients between TBF and visceral fat (0.13) and TBF and liver fat (0.17) were also low, indicating that additional adjustment for TBF does not lead to multicollinearity.

Lastly, to examine which component most strongly contributed to the associations of the DHD-index with visceral fat or liver fat, we performed analyses in which we subsequently left out one component at the time and additionally adjusted for that component. We reasoned that a component has an important contribution to the association if the association attenuates towards the null after leaving that component out. Linearity of the DHD-index and its components with visceral fat and liver fat was checked by adding a quadratic term to the main multivariable model and visual inspection of scatter plots.

We performed several subgroup analyses. Because TBF and VAT and HTGC may differ greatly between persons with and without obesity [29], between men and women, and between pre- and post-menopausal women [30], we stratified the multivariable model not including TBF by these variables. We additionally stratified the multivariable models for liver fat by the rs738409 single nucleotide polymorphism in the patatin-like phospholipase domain containing 3 (PNPLA3) gene, because carriers might have, in part, genetically induced liver fat which might be less strongly related to dietary habits [31].

Due to a skewed distribution of HTGC, we used the natural logarithm of this variable in the analyses. For interpretation of the results, we back transformed the regression coefficients of HTGC towards a ratio with 95% confidence interval, which is associated with a 10 points higher DHD-index. Such ratio, for example a ratio of 1.2, can be interpreted as each 5 points higher DHD-index being associated with a 1.2-fold increased HTGC, which would reflect an increase in liver fat from, for example, 5–6%. Regression coefficients of TBF represent an absolute difference in TBF in % per 10 points higher DHD-index, and those of VAT an absolute difference in VAT in cm^2^.

As participants with diabetes might have altered their diet as a result of the diagnosis, we repeated all analyses excluding participants with a medical history of diabetes mellitus.

We performed all analyses using STATA statistical Software (Statacorp, College Station, Texas, USA), version 14.

## Results

A total of 6671 participants were included in the NEO study between September 2008 and October 2012. For the analyses with TBF as an outcome, we excluded participants without a body fat assessment (*n* = 31), implausible energy intake (*n* = 62) or missing energy intake (*n* = 4), an incomplete FFQ (*n* = 23) or missing data on smoking (*n* = 7), education (*n* = 62), ethnicity (*n* = 8) or physical activity (*n* = 114), leaving a total of 6630 participants.

For the analyses on VAT, we additionally excluded participants without an MRI of the abdomen (*n* = 3912), which was performed in a random subsample of participants without contraindications. As a result, those who underwent the MRI have a slightly lower BMI (25.9 versus 26.6 kg/m^2^) and slightly less often a medical history of cardiovascular disease (4.1% versus 6.6%) than those without MRI. All other characteristics were similar. The total study population for the analyses on VAT contained 2450 participants.

For the analyses with HTGC as an outcome, we additionally excluded participants without HTGC measurement (*n* = 464). The majority of this missing values was due to technical failure, as it was not possible to check the spectra and repeat the measurement in the limited time available per participant. However, the failure rate of the MR spectroscopy was not related to age (55 years for participants with HTGC measurement versus 56 years for participants without HTGC measurement), sex (47% men versus 48% men), BMI (25.9 versus 26.2 kg/m^2^), VAT (89 versus 94 cm^2^) or the DHD-index (59.5 versus 58.8 points). Lastly, we excluded participants who consumed 40 g of alcohol or more (four standard glasses) per day (*n* = 176) and one participant for whom the natural logarithm of HTGC could not be calculated, leaving a total of 1809 participants.

The baseline characteristics of the total population for the analyses on TBF stratified by tertiles of the DHD-index are shown in Table 1. Participants in the highest tertile and thus who adhere the most to the dietary guidelines, more often had a high education, were female and nonsmoker.

**Table 1:** Baseline characteristics stratified by tertiles of the DHD-index in of participants of the Netherlands Epidemiology of Obesity study, men and women between 45 and 65 years of age

	DHD-index		
	Tertile 1 (19.6–64.3)	Tertile 2 (≥64.3–77.8)	Tertile 3 (≥77.8–119.1)
Demographic variables			
Age (year)	55 (7)	56 (6)	56 (5)
Sex (% men)	55.6	43.7	31.0
Ethnicity (% white)	94.5	95.4	94.9
Education level (% high)^a^	37.1	46.2	54.8
Tobacco smoking (% current)	28.8	13.1	6.6
Menopausal state (% post in women)^b^	56.6	57.4	65.4
Physical activity in leisure time (MET h/week)	26.3 [12.0–44.3]	30.0 [16.9–50.5]	32.7 [18.5–52.3]
Dietary variables			
DHD-index	54.4 (7.8)	71.0 (4.0)	87.3 (6.7)
Fruit and vegetable intake (g/d)	235 (142)	322 (154)	419 (142)
Alcohol intake (g/d)	18.2 [4.0–31.3]	9.2 [2.6–20.8]	7.5 [2.0–14.3]
Energy restricted diet (%)	9.4	11.6	14.5
Basal metabolic rate (MJ/d)	6.6 (1.2)	6.4 (1.1)	6.0 (0.9)
Body fat measures			
BMI (kg/m^2^)	26.9 (4.8)	26.5 (4.5)	25.5 (3.9)
Total body fat (%)			
Men	25.7 (6.6)	25.1 (6.0)	23.7 (5.3)
Women	37.4 (7.3)	37.3 (6.6)	36.3 (5.7)
Visceral adipose tissue (cm^2^)^c^			
Men	123.7 (67.4)	114.6 (59.1)	104.0 (51.4)
Women	69.7 (44.2)	68.3 (46.2)	63.3 (34.2)
Hepatic triglyceride content (%)^d^			
Men	4.9 [2.0–13.1]	3.7 [2.2–7.8]	3.2 [1.9–6.2]
Women	2.2 [1.2–5.8]	1.8 [1.1–5.1]	1.6 [1.1–3.3]
Waist circumference (cm)			
Men	99.7 (12.2)	98.5 (11.2)	96.0 (9.7)
Women	88.9 (14.3)	87.8 (13.2)	85.7 (11.0)
CVD risk factors			
CVD (%)	5.8	5.9	5.0
Lipid lowering medication (%)	11.2	10.6	9.3
Total cholesterol (mmol/L)	5.7 (1.1)	5.7 (1.1)	5.6 (0.9)
Fasting triglycerides (mmol/L)	1.4 (1.1)	1.3 (0.9)	1.1 (0.6)
HDL cholesterol (mmol/L)	1.5 (0.5)	1.5 (0.4)	1.6 (0.4)

Results are based on analyses weighted toward the BMI distribution of the general population (*n* = 6360). Data are shown as mean (standard deviation), median [interquartile range], or percentage

*BMI* body mass index, *CVD* cardiovascular disease, *HDL* high-density lipoproteins, *MET* metabolic equivalent of task

^a^Low education: none, primary school, or lower vocational education as highest level of education

^b^Proportion menopausal state only estimated in women (*n* = 3352)

^c^Mean VAT only calculated in persons with VAT measurement (*n* = 2450)

^d^Mean HTGC only calculated in persons with HTGC measurement (*n* = 1809)

### Dutch Healthy Diet Index in relation to total body fat

**Fig. 1:**
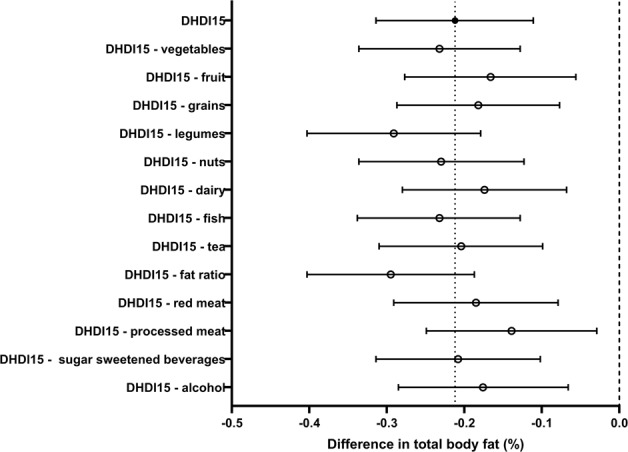
Association between 10 points on the Dutch Healthy Diet Index and total body fat when leaving one component out at the time (*n* = 6361), adjusted for sex, age, smoking status, education, ethnicity, basal metabolic rate, menopausal state, energy restricted diet, total energy intake, and component left out. Results are based on analyses weighted toward the body mass index distribution of the general population. DHDI Dutch Healthy Diet Index, PA physical activity, SFA saturated fatty acids, TFA trans fatty acids, TBF total body fat

After adjustment for potential confounding factors, 10 points higher on the DHD-index was associated with 0.2% less TBF (95% CI −0.3; −0.1%) (Table 2). Of all components, leaving out the processed meat component attenuated the association, as did dairy and fruit (Fig. 1). Results were similar in men and women (data not shown).

**Table 2:** Difference in measure of body fat with 95% confidence intervals per 10 points higher on the DHD-index in participants of the Netherlands Epidemiology of Obesity study, men and women between 45 and 65 years of age

	Total body fat (*n* = 6360)	Visceral adipose tissue (*n* = 2450)	Hepatic triglyceride content (*n* = 1809)
	Difference in TBF (%) (95% CI)	Difference in VAT (cm^2^) (95% CI)	Relative change in HTGC (95% CI)
Crude			
Total	0.5 (0.3; 0.7)	−6.7 (−8.5; −4.9)	0.90 (0.86; 0.94)
Men	−0.6 (−0.8; −0.4)	−5.6 (−8.6; −2.7)	0.93 (0.87; 1.00)
Women	−0.4 (−0.6; −0.2)	−1.6 (−3.5; 0.4)	0.93 (0.87; 0.99)
Multivariable^a^			
Total	−0.2 (−0.3; −0.1)	−3.2 (−4.7; −1.8)	0.92 (0.88; 0.96)
Men	−0.3 (−0.4; −0.1)	−4.3 (−6.9; −1.8)	0.93 (0.88; 1.00)
Women	−0.2 (−0.3; −0.1)	−2.2 (−3.7; −0.6)	0.91 (0.85; 0.96)
Multivariable + TBF			
Total		−2.3 (−3.5; −1.0)	0.94 (0.90; 0.98)
Men		−2.3 (−4.4; −0.2)	0.96 (0.91; 1.00)
Women		−1.4 (−2.8; −0.1)	0.92 (0.87; 0.98)

Results are based on analysis weighted toward the body mass index distribution of the general population

*CI* confidence interval, *DHDI* Dutch Healthy Diet Index, *TBF* total body fat, *VAT* visceral adipose tissue

^a^Adjusted for age, total energy intake, smoking, education, ethnicity, basal metabolic rate, menopause, and energy restricted diet

After stratification by BMI, results were similar for both groups (Supplemental Table 1). The association between the DHD-index and TBF was somewhat stronger in postmenopausal women than in premenopausal women (Supplemental Table 1).

### Dutch Healthy Diet Index in relation to visceral adipose tissue

**Fig. 2:**
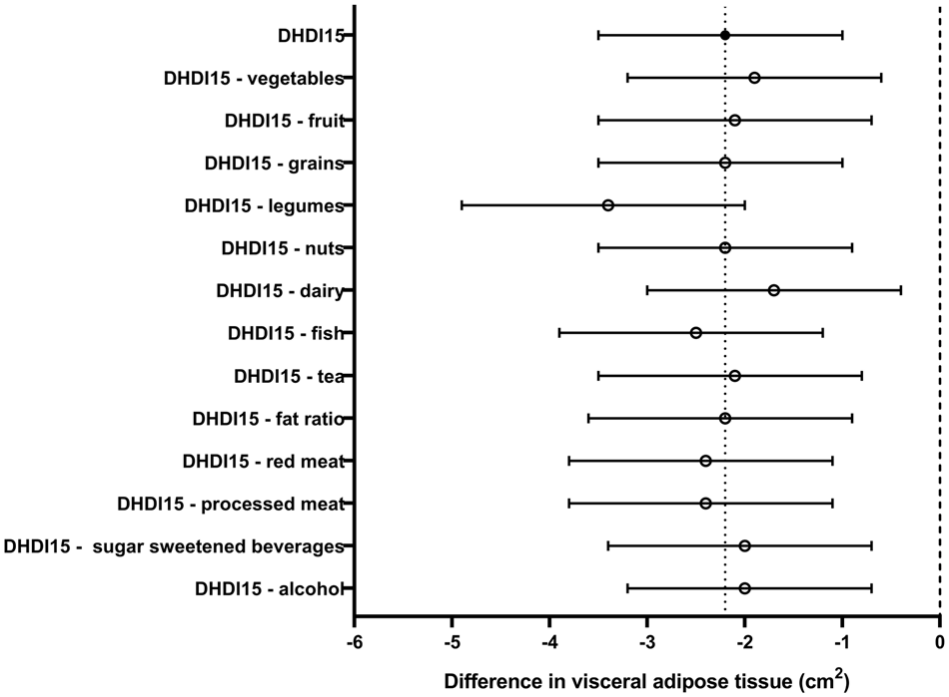
Association between 10 points on the Dutch Healthy Diet Index and visceral adipose tissue when leaving one component out at the time (*n* = 2449), adjusted for sex, age, smoking status, education, ethnicity, basal metabolic rate, menopausal state, energy restricted diet, total energy intake, total body fat, and component left out. Results are based on analyses weighted toward the body mass index distribution of the general population. DHDI Dutch Healthy Diet Index, PA physical activity, SFA saturated fatty acids, TFA trans fatty acids, VAT visceral adipose tissue

After adjustment for potential confounding factors and TBF, the DHD-index was inversely associated with VAT (−2.2 cm^2^ per 10 points higher on the DHD-index, 95% CI −3.5; −1.0 cm^2^) **(**Table 2). Of all components, leaving out the dairy component of the DHD-index slightly attenuated the association (Fig. 2). Results were similar in men and women (data not shown).

When stratified by BMI, the association between the DHD-index and VAT was similar in participants with or without obesity and in post- and pre-menopausal women (Supplemental Table 1).

### Dutch Healthy Diet Index in relation to hepatic triglyceride content

**Fig. 3:**
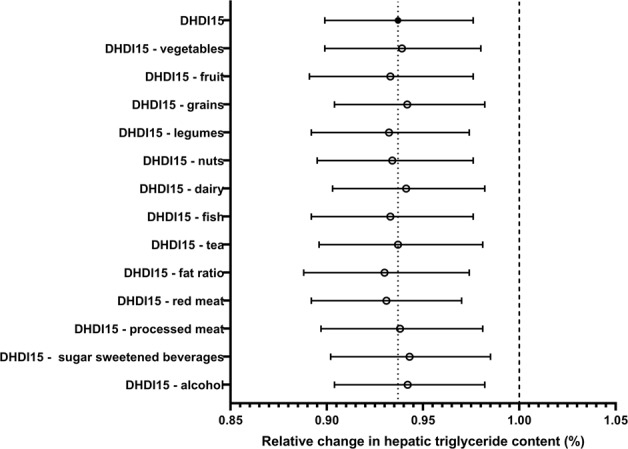
Association between 10 points on the Dutch Healthy Diet Index and hepatic triglyceride content when leaving one component out at the time (*n* = 1809), adjusted for sex, age, smoking status, education, ethnicity, basal metabolic rate, menopausal state, energy restricted diet, total energy intake, total body fat, and component left out. Results are based on analyses weighted toward the body mass index distribution of the general population. DHDI Dutch Healthy Diet Index, HTGC hepatic triglyceride content, PA physical activity, SFA saturated fatty acids, TFA trans fatty acids

After adjustment for potential confounding factors and TBF, 10 points higher on the DHD-index was associated with less liver fat (0.94 times, 95% CI 0.90; 0.98) (Table 2). Leaving out components did not alter the association (Fig. 3). Associations were comparable for men and women (data not shown). Associations between the DHD-index and liver fat were comparable in participants with or without obesity (Supplemental Table 1). When stratified by menopausal state, associations were only present in postmenopausal women. Associations were similar in both carriers and noncarriers of the PNPLA3 risk allele (Supplemental Table 1).

## Discussion

In this population-based study of middle-aged men and women, we aimed to study the association between adherence to the Dutch Guidelines for a Healthy Diet 2015 and TBF, visceral fat and liver fat, as assessed with bio impedance analysis, MRI, and ^1^H-MRS. After adjustment for potential confounding factors, a higher score on the DHD-index, and therefore a greater adherence, was associated with less TBF, less visceral fat, and less liver fat. Associations with visceral fat and liver fat remained present after adjustment for TBF, indicating specific associations with visceral fat and liver fat rather than with overall adiposity. When leaving all the thirteen components out one by one to examine which component contributes the most to the association, all components seemed similarly important. No clear overall differences were observed between BMI categories, post- and pre-menopausal women or between carriers and noncarriers of the PNPLA3 risk allele.

Several previous studies have shown associations between diet quality indices with a moderate protective effect regarding multiple health outcomes, as reduced risks of cardiovascular disease and mortality [15]. In a meta-analysis, adherence to high-quality diets as assessed by the (Alternative) HEI and the Dietary Approaches to Stop Hypertension were associated with decreased risks of all-cause mortality (22%), CVD (22%), cancer (15%), and of type 2 diabetes (22%) [32]. Although the 2015 DHD-index is relatively new, a higher adherence to the 2015 Dutch Guidelines for a Healthy Diet has been associated with a decreased risk of stroke, chronic obstructive pulmonary disease, colorectal cancer, and all-cause mortality [33].

In another recent systematic review on diet quality indices in relation to measures of obesity it has been shown that adherence to the HEI, the Dietary Guidelines for Americans Index and the Dietary Guideline Index were associated with either lower BMI or waist circumference [34]. Moreover, in a recent meta-analysis, healthy dietary patterns were inversely related to both visceral fat and subcutaneous fat [35], although most studies did not adjust for TBF and therefore the associations might not be specific for visceral fat and subcutaneous fat. Dietary intake of fiber and calcium was inversely related with visceral fat, and there was a positive relation of unhealthy dinner-type dietary patterns and consumption of alcohol and fructose with visceral fat [35]. Our study contributes to this knowledge by showing that adherence to dietary guidelines for a healthy diet as a whole was not only associated with TBF, but also specifically with visceral fat and liver fat. Our findings thereby suggest that next to TBF, visceral fat, and liver fat may mediate the previous observed associations of diet indices with cardiometabolic risk.

Improvement of diet quality in terms of an increase in the (Alternative) HEI 2010, the alternate Mediterranean Diet Score and the Dietary Approaches to Stop Hypertension score have been associated with decreased weight gain, especially in people with a BMI over 25 kg/m^2^ [36]. This corresponds with our findings that showed that the association between the DHD-index and VAT was slightly stronger in people with a BMI over 30 kg/m^2^, but not for liver fat.

A study on the HEI-2010 in young Americans has also demonstrated an inverse association with body fatness in men, after taking level of physical activity into account [37].

Strengths of this study are the sample size and the extensive phenotyping, allowing adjustment for multiple potential confounding factors and investigation of multiple subgroup analyses. Moreover, we directly assessed VAT and hepatic triglyceride by MRI and ^1^H-MRS in a relatively large subsample of the study population. The DHD-index is a measure of adherence to the current (2015) Dutch dietary guidelines and reflects the whole diet as it includes multiple food group based components. Multiple improvements have been made compared with the previous 2006 guidelines and DHD-index. For example, fruit juices are now no longer included in the fruit component bur rather in the sweetened beverages component, and whereas the previous guidelines focused on saturated fat without taking the source into account, the new index includes a component on the solid to liquid fat ratio.

A limitation of our study is that dietary intake of food products is measured with a self-administered FFQ, making it prone to measurement error. Potential social desirability might have overestimated the average score. Although this might have affected associations with TBF, it is less likely that this would affect associations with visceral fat or liver fat because people are not aware of the amount of visceral fat or liver fat they have. Moreover, TBF has been estimated using BIA with the Tanita FF BIA system. Although it has been suggested that FF BIA might give an overestimation of the amount of fat mass [38], another study found a strong correlation (*r* = 0.84) between foot-to-foot and hand-to-foot BIA with regards to TBF percentages [18]. Furthermore, a strong correlation (*r* = 0.89) was also found in a study comparing resistance measurements provided by FF BIA with measurements from dual-energy X-ray absorptiometry and underwater weighing [39]. In addition, the population of this study predominantly consisted of Caucasian, middle-aged participants, so results should be confirmed in other age and ethnic groups. However, a large prospective cohort study conducted in the South-eastern part of the United States showed that associations between adherence to the Dutch Guidelines for Americans as assessed by the HEI-2010 were similar between African-Americans and whites [40]. As the FFQ used in our study did not contain complete information on certain food items, we had to make several assumptions in order to calculate the 2015 DHD-index. For example, we could not make a distinction between salted and unsalted nuts. As a result, salted nuts and beer nuts are now included in this component, which could have influenced the results. Moreover, the wholegrain component is now only based on breakfast cereals, which may therefore result in measurement error. Lastly, the observational cross-sectional design of this study precludes causal inference.

Whereas the associations with visceral fat and liver fat content may seem weak, it must be noted that an increase of 10 points on the DHD-index can be easily accomplished and the results might therefore be considerably relevant in general practice. For example, consumption of one apple and one cup of broccoli per day extra adds up to a 10 points higher DHD-index, which was associated with more than 2 cm^2^ less visceral fat. As previous results has shown that visceral fat and liver have been associated with insulin resistance, metabolic risk factors, and cardiovascular disease [3–6], adherence to the Dutch Guidelines for a Healthy Diet might ultimately be associated with a decreased risk of developing insulin resistance or cardiovascular disease, although direct and exact translation to disease risk remains difficult.

In conclusion, in this population-based study in middle-aged men and women, adherence to the Dutch Guidelines for a Healthy Diet from 2015 as assessed by the DHD-index, was associated with less TBF, but also specifically with less visceral fat and liver fat. These associations do not seem driven by one component in particular, indicating the importance of an overall healthy lifestyle to prevent cardiometabolic disorders. These findings might contribute to better understanding of the mechanisms underlying associations between dietary habits and cardiometabolic diseases. Future intervention studies are therefore needed to assess whether, and to what extent, changes in a person’s lifestyle can specifically influence visceral fat and liver fat and thereby reduce the risk of cardiometabolic diseases.

## Supplementary information

Supplemental table 1
